# Esophagitis Dissecans Superficialis: An Uncommon Endoscopic Finding

**DOI:** 10.7759/cureus.113760

**Published:** 2026-07-31

**Authors:** Giovanni Francisco Perez Roa, Yoeli M Escandon-Espinoza, Katia Daniela Lopez Garcia

**Affiliations:** 1 Gastrointestinal Endoscopy, Hospital Regional de Alta Especialidad Bicentenario de la Independencia, Instituto de Seguridad y Servicios Sociales de los Trabajadores del Estado (ISSSTE), Mexico City, MEX

**Keywords:** autoimmune bullous dermatoses, barium esophagogram, dysphagia, esophagitis dissecans superficialis, sloughing esophagitis, upper gastrointestinal endoscopy

## Abstract

Esophagitis dissecans superficialis (EDS), also known as sloughing esophagitis, is a rare and frequently underrecognized esophageal disorder characterized by superficial sloughing of the esophageal mucosa. Its variable clinical presentation and overlap with structural esophageal abnormalities may result in misdiagnosis and unnecessary diagnostic or therapeutic interventions. We report a case of a 25-year-old woman with no history of chronic degenerative, autoimmune, or atopic disease who presented with abrupt retrosternal chest pain, recurrent vomiting, progressive esophageal dysphagia, halitosis, hematemesis, and marked unintentional weight loss. Initial evaluation showed a double-lumen appearance on barium esophagography and an upper endoscopic appearance initially interpreted as a large pharyngoesophageal diverticulum. Repeat endoscopy subsequently revealed extensive longitudinal sloughing and detachment of the esophageal mucosa, supporting the diagnosis of EDS. Histopathologic evaluation demonstrated a submucosal blistering lesion infiltrated by eosinophils, without evidence of malignancy or dysplasia. These findings raised suspicion for esophageal bullous pemphigoid; however, direct immunofluorescence was unavailable, and an autoimmune bullous disorder could not be definitively confirmed. Additional computed tomography imaging showed no evidence of esophageal disruption, mediastinal collection, pneumomediastinum, malignancy, or another structural abnormality. Severe oral intolerance, recurrent vomiting, persistent dysphagia, and marked nutritional compromise led the nutrition support team to select temporary total parenteral nutrition as an individualized strategy pending clinical and endoscopic reassessment. The severe mucosal involvement and suspected autoimmune bullous component prompted empiric treatment with intravenous corticosteroids. The patient achieved clinical stabilization, and outpatient endoscopic reassessment was scheduled. In the absence of follow-up endoscopic findings, no conclusions regarding mucosal healing or treatment efficacy could be drawn. This case highlights the importance of careful clinical, endoscopic, radiologic, and histopathologic correlation in severe or atypical presentations of EDS, particularly when the findings mimic a structural esophageal disorder.

## Introduction

Esophagitis dissecans superficialis (EDS), also referred to as sloughing esophagitis or desquamative esophagitis, is a rare esophageal disorder characterized by detachment of large fragments of superficial squamous mucosa into the esophageal lumen [1-3]. Its true incidence is likely underestimated because symptoms are often non-specific and the diagnosis is primarily endoscopic. A retrospective analysis of 21,497 esophagogastroduodenoscopies reported an incidence of approximately 0.03%, supporting its rarity in routine clinical practice [4-6].

The etiology and pathogenesis of EDS remain incompletely understood. Although some cases are idiopathic, reported associations include medications such as bisphosphonates, nonsteroidal anti-inflammatory drugs, potassium chloride, and psychoactive agents; mucosal irritants such as hot beverages, smoking, and chemical injury; physical trauma; esophageal motility disorders; connective tissue diseases; and autoimmune bullous dermatoses [1,4,6-8]. EDS has been described more frequently in older, debilitated, or polymedicated patients; however, atypical presentations in younger individuals may occur [2,4,6].

Endoscopically, EDS typically appears as whitish strips, sheets, or columns of easily detachable mucosa, often with macroscopically normal underlying esophageal tissue [4,6,7]. Histopathologic findings reported in the literature may include detached superficial epithelium, parakeratosis, intraepithelial splitting, superficial epithelial necrosis, and minimal inflammation [4,6,7]. These findings are not pathognomonic and should be interpreted in conjunction with the clinical and endoscopic presentation. In selected cases, histologic findings may overlap with those of autoimmune bullous disorders [8-12].

We present a case of a young woman with severe progressive dysphagia, hematemesis, marked nutritional compromise, and a double-lumen-like esophageal appearance initially interpreted as a structural abnormality. This case highlights the diagnostic challenges of EDS and the importance of considering autoimmune bullous involvement in selected patients, even in the absence of obvious mucocutaneous bullous lesions.

## Case presentation

A 25-year-old woman with no personal history of chronic degenerative, autoimmune, or atopic disease presented with progressive esophageal symptoms that had begun approximately two months before admission. She denied tobacco use and chronic medication exposure. Her initial manifestations consisted of abrupt retrosternal chest pain associated with nausea and recurrent vomiting. She was initially evaluated at an outside healthcare institution, where a cardiac etiology was excluded, and symptomatic treatment with double-dose proton pump inhibitor therapy and a prokinetic agent was prescribed without clinical improvement. Initial laboratory tests obtained at presentation were largely unremarkable, except for mild hyponatremia and elevated blood urea nitrogen. Laboratory findings are summarized in Table 1, including units of measurement and corresponding normal reference values.

**Table 1:** Laboratory findings at initial presentation. *Glucose interpretation depends on fasting status, which was not specified. **Prothrombin time reference values vary according to the reagent and laboratory method; interpretation should be adjusted to the institutional reference range. INR was within normal limits. INR: international normalized ratio

Laboratory parameter (units)	Result	Normal reference value
Leukocytes (cells/µL)	8,280	4,000-11,000
Neutrophils (cells/µL)	3,650	1,500-7,500
Hemoglobin (g/dL)	12.8	12-16
Mean corpuscular volume (fL)	86	80-100
Platelets (cells/µL)	317,000	150,000-450,000
Glucose (mg/dL)	101	70-99*
Creatinine (mg/dL)	0.74	0.50-1.10
Blood urea nitrogen (mg/dL)	28	7-20
Sodium (mEq/L)	132	135-145
Potassium (mEq/L)	3.79	3.5-5.1
Magnesium (mg/dL)	2.2	1.7-2.4
Prothrombin time (s)	16.5	11-13.5**
International normalized ratio	1.01	0.8-1.2

Over the following weeks, the patient developed progressive esophageal dysphagia, halitosis, persistent chest pain related to swallowing, and recurrent vomiting of gastric and bilious contents. She also reported one episode of large-volume hematemesis. Her symptoms progressively limited oral intake and led to significant unintentional weight loss of approximately 13 kg over two months. Because of persistent dysphagia and poor oral tolerance, a nasogastric feeding tube was placed at an outside institution.

**Figure 1:**
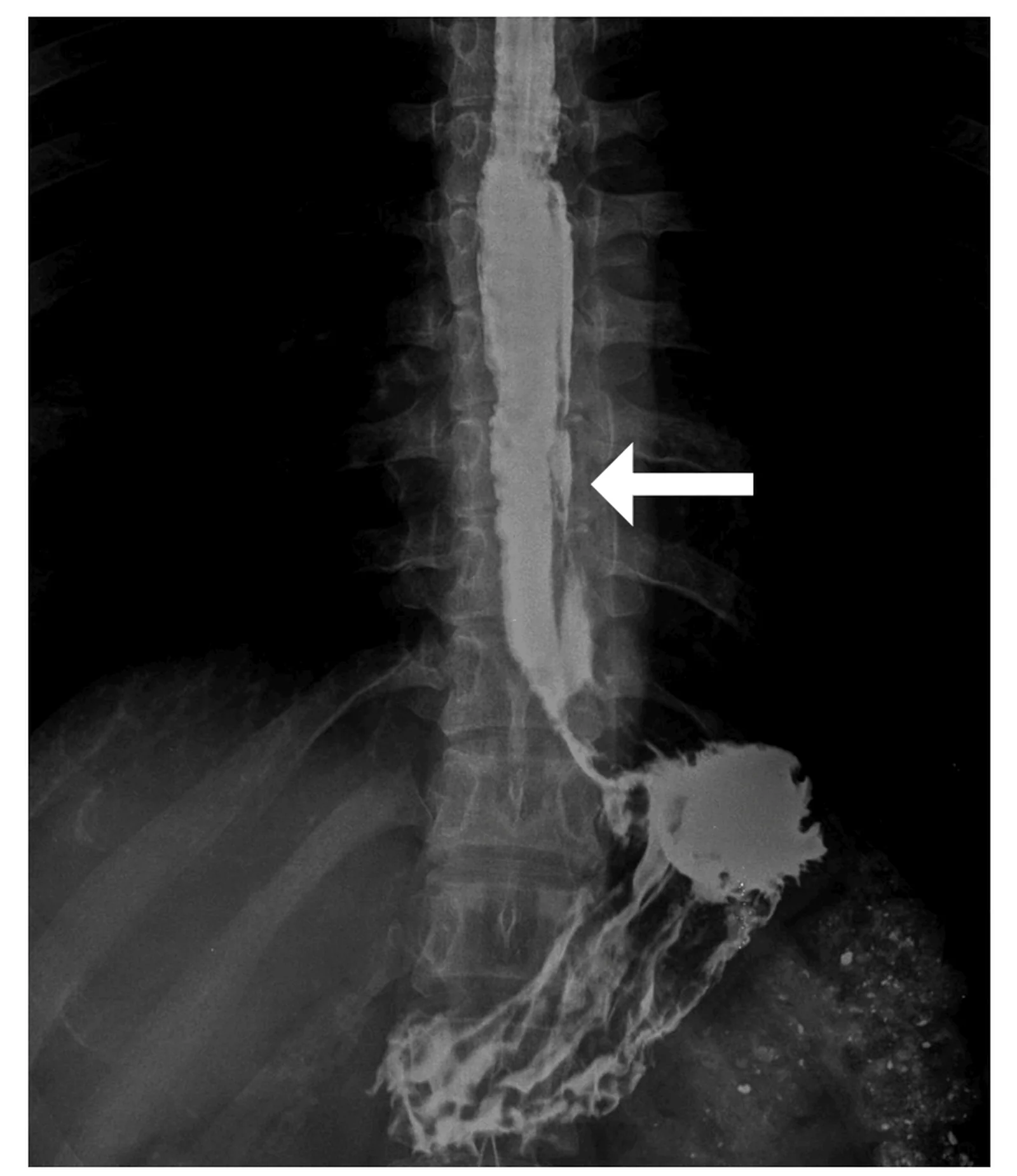
Initial barium esophagogram demonstrating a double-lumen appearance of the esophagus. Contrast esophagography showing a longitudinal linear filling defect involving the mid and distal esophagus highlighted by the white arrow, producing a characteristic double-lumen configuration.

**Figure 2:**
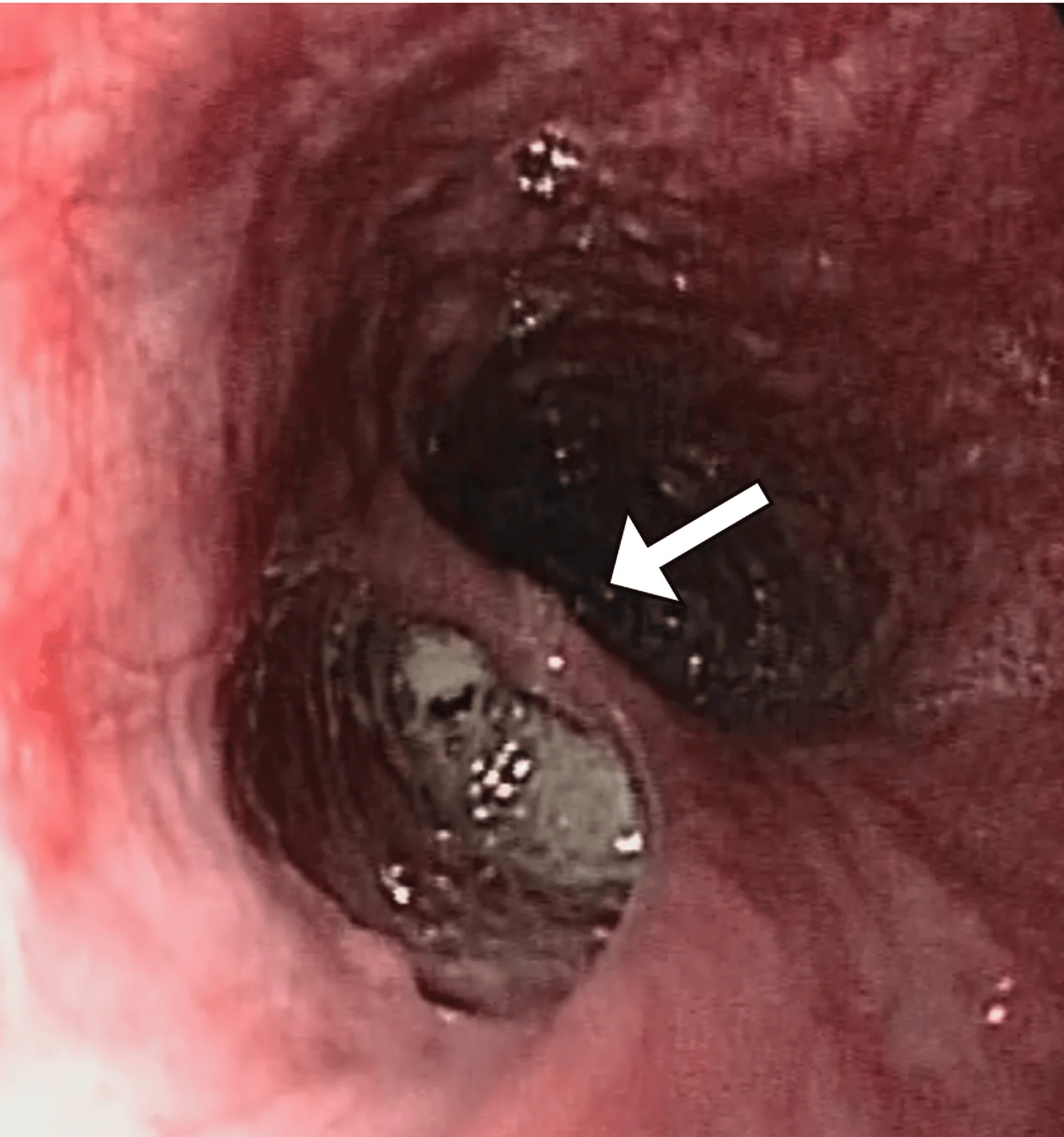
Initial upper endoscopy showing a double-lumen appearance of the esophagus. Upper endoscopy performed at an outside institution demonstrating a double-lumen configuration initially interpreted as a structural abnormality suggestive of an esophageal diverticulum. The white arrow highlights the area of interest corresponding to the apparent double-lumen configuration. Based on these findings and the subsequent lack of clinical-endoscopic correlation, the patient was referred for further evaluation and repeat endoscopy at our center.

A barium esophagogram demonstrated a longitudinal linear filling defect involving the middle and distal esophagus, producing a double-lumen configuration (Figure 1). Initial upper endoscopy performed at an outside institution showed a double-lumen-like configuration that was initially interpreted as a large pharyngoesophageal diverticulum measuring approximately 15 cm, together with ulcerative gastric findings (Figure 2). However, the patient’s clinical course and the imaging-endoscopic correlation were not fully consistent with a simple structural diverticular disorder; therefore, she was referred for further evaluation.

On admission, the patient was hemodynamically stable and adequately hydrated. Physical examination revealed cutaneous pallor, normal cardiopulmonary findings, and a soft, non-tender abdomen with preserved bowel sounds. No active bleeding, respiratory compromise, systemic infection, or mucocutaneous bullous lesions were identified.

**Figure 3:**
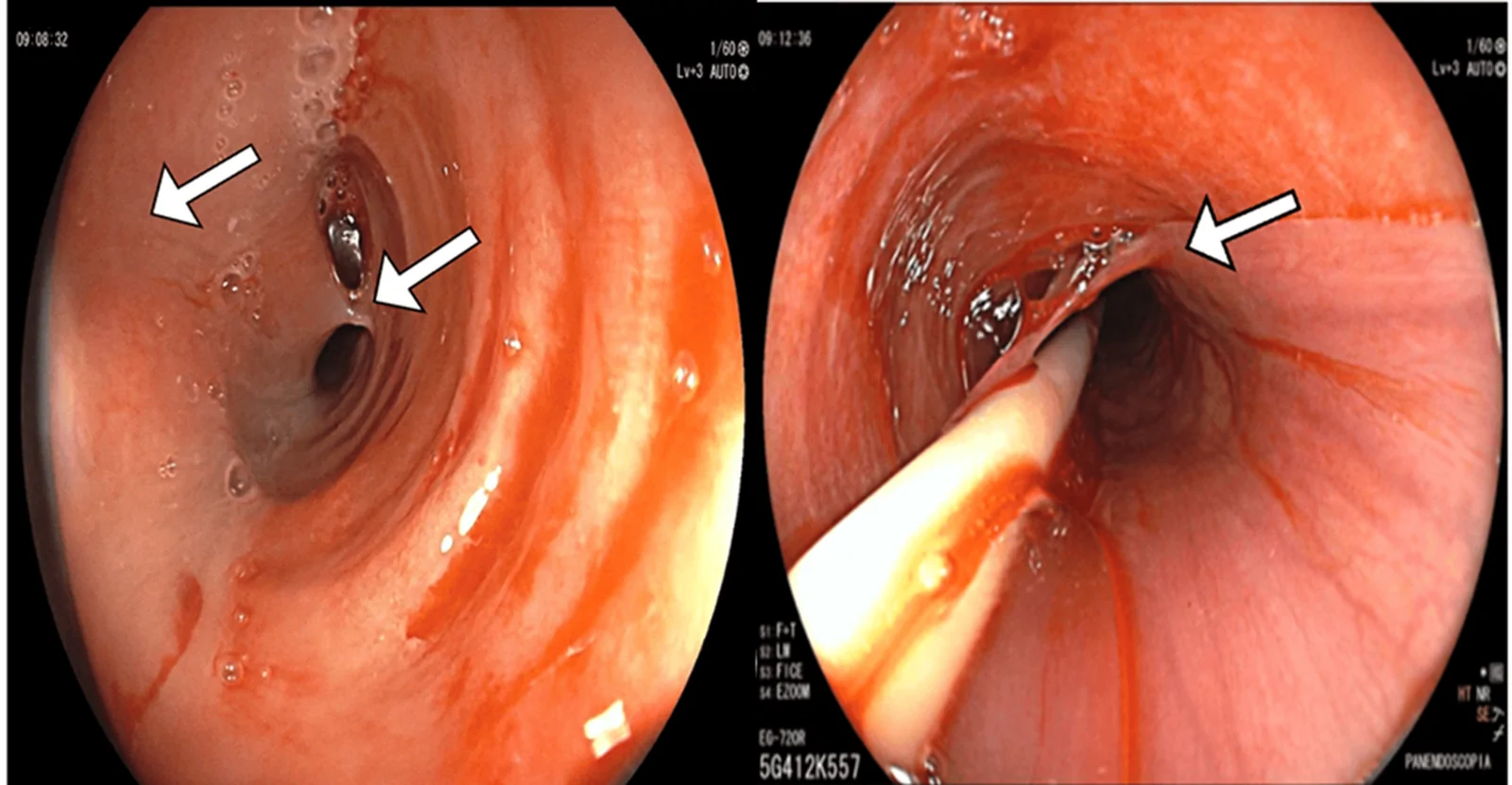
Endoscopic findings consistent with esophagitis dissecans superficialis. Repeat upper endoscopy performed at our institution demonstrated extensive longitudinal sloughing of the esophageal mucosa with characteristic vertical whitish strips highlighted by white arrows, consistent with esophagitis dissecans superficialis. These findings contrast with the initial endoscopic appearance and support the final diagnosis.

Repeat upper endoscopy revealed extensive longitudinal sloughing and detachment of the esophageal mucosa, extending for approximately 15 cm, with a double-lumen-like appearance and distal esophageal orifices of similar morphology (Figure 3). These findings were considered atypical for simple diverticular disease and raised the differential diagnosis of EDS, spontaneous partial mucosal laceration, and autoimmune bullous involvement of the esophagus. Multiple biopsies were obtained.

**Figure 4:**
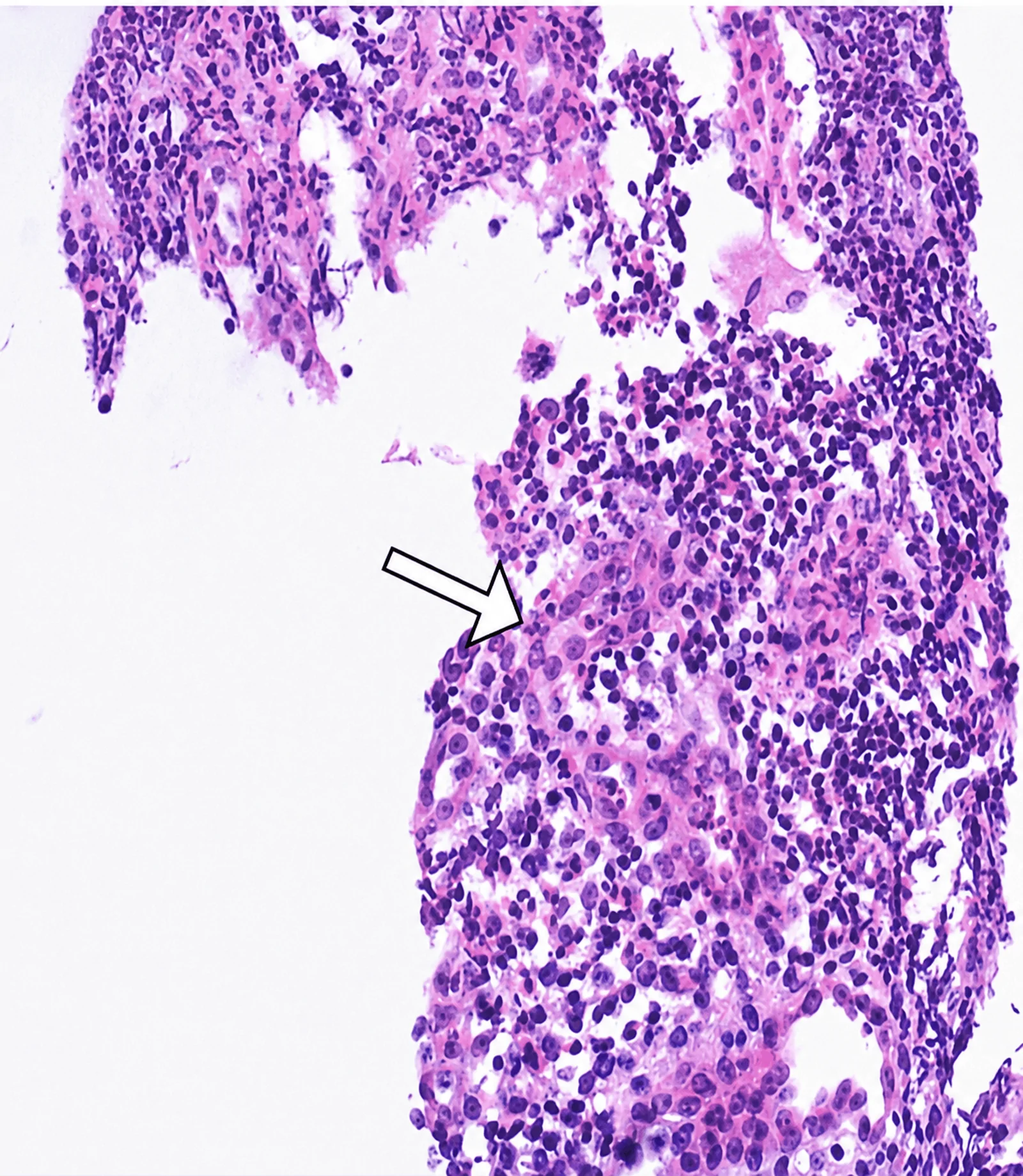
Histopathologic features of esophagitis dissecans superficialis. Histologic section showing a submucosal blistering lesion infiltrated by eosinophils. The arrow highlights the area of blistering change. These findings raised suspicion for esophageal bullous pemphigoid; however, immunofluorescence was recommended for diagnostic confirmation (H&E stain).

Histopathologic evaluation demonstrated a submucosal blistering lesion infiltrated by eosinophils. The adjacent mucosa showed edema and a chronic perivascular inflammatory infiltrate, while the macroscopically preserved mucosa exhibited vascular congestion. No malignancy or dysplasia was identified. These findings raised suspicion for esophageal bullous pemphigoid, and immunofluorescence was recommended for diagnostic confirmation. However, direct immunofluorescence was unavailable; therefore, an autoimmune bullous disorder remained presumptive rather than confirmed (Figure 4).

**Figure 5:**
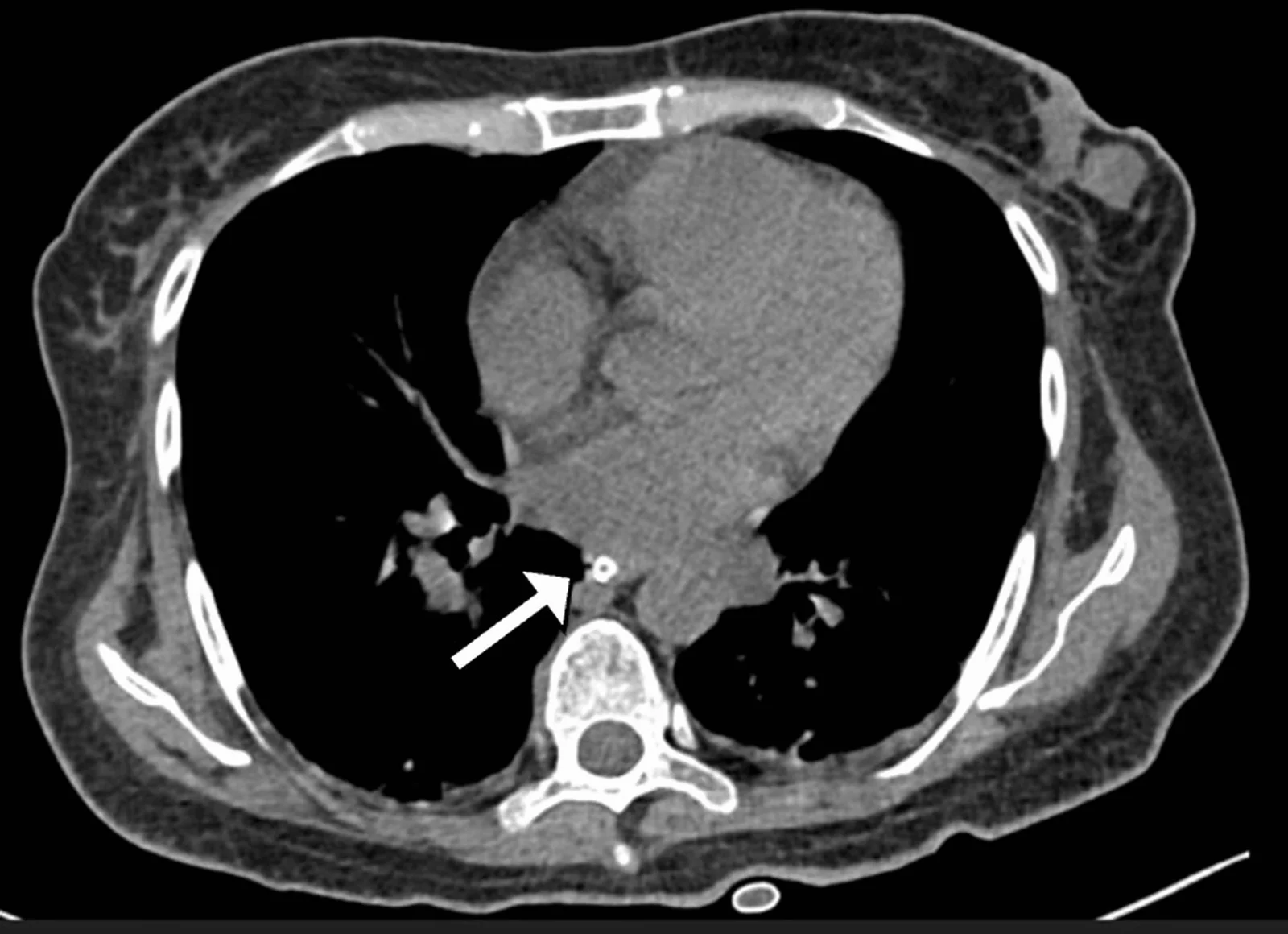
Axial computed tomography image of the thorax. The white arrow highlights the radiopaque nasogastric tube along the expected esophageal tract. No clear evidence of esophageal disruption, mediastinal collection, or pneumomediastinum is observed.

Additional imaging was performed to exclude complications and alternative diagnoses. Computed tomography (CT) of the neck and thorax showed the nasogastric tube along the expected esophageal tract, without clear evidence of esophageal disruption, collections, or pneumomediastinum (Figure 5). CT of the chest, abdomen, and pelvis did not demonstrate evidence of malignancy or another structural disease. CT was used to assess complications and alternative structural diagnoses, not as a primary diagnostic tool for EDS. The overall clinical, endoscopic, radiologic, and histopathologic correlation supported the diagnosis of EDS.

Severe oral intolerance, recurrent vomiting, persistent dysphagia, and approximately 13 kg of unintentional weight loss led the nutrition support team to select temporary total parenteral nutrition (TPN) as an individualized strategy pending clinical and endoscopic reassessment. Although enteral nutrition is generally preferred whenever feasible, poor tolerance and severe esophageal mucosal injury limited its continued use. A central venous catheter was placed, and the patient and her caregivers received training in catheter care. Laboratory monitoring was scheduled every 15 days to assess metabolic and biochemical complications, together with surveillance for catheter-related complications.

The severe mucosal involvement and suspected autoimmune bullous component prompted empiric treatment with intravenous methylprednisolone at an initial dose of 40 mg daily. The dose was subsequently tapered during hospitalization. Direct immunofluorescence and follow-up endoscopic findings were unavailable; therefore, neither the autoimmune etiology nor a steroid-related treatment response could be confirmed.

After clinical stabilization and training for home parenteral nutrition and central venous catheter care, she was discharged with strict nil per mouth status until further medical evaluation, continuation of parenteral nutrition at home, intravenous hydration supplementation, and intravenous methylprednisolone 16 mg every 24 h, equivalent to 20 mg of prednisone, until reassessment. Follow-up laboratory monitoring every 15 days and repeat outpatient endoscopic evaluation were scheduled to guide further therapeutic decisions. At the time of manuscript revision, the results of the scheduled follow-up endoscopy were not available.

## Discussion

EDS is an infrequent and underdiagnosed entity in routine clinical practice [4,6]. Although it is often described as a benign condition, its clinical expression is heterogeneous, ranging from incidental endoscopic findings to debilitating symptoms such as dysphagia, odynophagia, chest pain, nausea, vomiting, hematemesis, and weight loss [4,6,7,13]. In rare cases, patients may even expectorate or vomit fragments of sloughed esophageal mucosa [4,5].

The typical demographic profile includes older, chronically debilitated, or polymedicated patients; however, EDS may also occur in younger individuals without classic risk factors [2,4,6]. Younger adult cases have also been reported, including a 49-year-old man with EDS identified after thermal injury [1]. Nevertheless, reports in patients as young as the present 25-year-old patient remain uncommon. This case is distinguished by the patient’s young age, the absence of classic risk factors or triggering medications, the severe degree of nutritional compromise, and the absence of clinically evident mucocutaneous bullous lesions. These characteristics contrast with the more commonly reported demographic and clinical profile, making the presentation atypical and diagnostically challenging [2,4,6].

The diagnosis of EDS is primarily based on endoscopic recognition. Proposed endoscopic criteria include sloughed esophageal mucosal strips longer than 2 cm, normal underlying mucosa, and the absence of adjacent ulceration or friability [6]. Although imaging studies are not routinely required, esophagographic findings may demonstrate linear filling defects, mucosal irregularity, edema, or strictures [5,6]. In our case, the double-lumen appearance on the barium esophagogram and the initial interpretation of a large pharyngoesophageal diverticulum created diagnostic uncertainty and mimicked a structural esophageal disorder.

The differential diagnosis of EDS is broad and includes Candida esophagitis, eosinophilic esophagitis, pill-induced esophagitis, caustic or thermal injury, lichen planus, squamous neoplasia, spontaneous mucosal laceration, and autoimmune bullous disorders [4,6-8]. Eosinophilic esophagitis was considered because of the eosinophilic infiltrate; however, the available histopathologic report did not provide a quantitative eosinophil count, and this diagnosis could not be assessed solely on the available histologic description. No malignancy or dysplasia was identified on histopathologic evaluation. Esophageal disruption, mediastinal collection, and pneumomediastinum were not evident on CT imaging. A structural diverticular disorder was considered initially, but repeat endoscopy and the overall clinical-endoscopic correlation were not consistent with a simple diverticulum.

Histopathologic evaluation may support the diagnosis of EDS but is often non-specific. Findings reported in the literature include prominent parakeratosis, intraepithelial splitting, superficial epithelial necrosis, and minimal inflammation [4,6,7]. These findings should be interpreted in conjunction with clinical and endoscopic features rather than as standalone diagnostic criteria. In our patient, the submucosal blistering lesion infiltrated by eosinophils raised concern for an autoimmune bullous process. Nevertheless, the absence of direct immunofluorescence prevented definitive confirmation of esophageal bullous pemphigoid.

The association between EDS and autoimmune bullous dermatoses has been well described [8]. Raque et al. first reported esophageal involvement in pemphigus vulgaris, and subsequent studies have documented a spectrum of endoscopic manifestations ranging from mild mucosal changes to classic EDS [9-11]. Although most reported cases present with concomitant mucocutaneous involvement, isolated esophageal disease has also been described [10,11]. This underscores the importance of maintaining a high index of suspicion even in the absence of dermatologic findings.

Endoscopic evaluation poses potential risks because of the fragility of the esophageal mucosa and the lack of standardized biopsy techniques. Galloro et al. highlighted the importance of obtaining tissue samples that include the basement membrane, recommending biopsies at the junction between the blister roof and floor and from adjacent mucosa [12]. In the present case, the blistering pattern and mucosal fragility required careful sampling and reinforced the need for a cautious endoscopic approach.

Laboratory findings in EDS are generally non-specific, and there is no established laboratory biomarker for diagnosis. Reported cases may show normal laboratory results, non-specific inflammatory changes, or abnormalities related to comorbidities, dehydration, nutritional compromise, bleeding, or associated conditions. In the present case, the initial laboratory findings were largely unremarkable except for mild hyponatremia and elevated blood urea nitrogen, which were not diagnostic of EDS. Therefore, normal or near-normal laboratory findings do not exclude EDS, and the diagnosis should rely primarily on endoscopic recognition supported by histopathologic and clinical correlation.

Currently, no standardized treatment for EDS exists, as many cases resolve spontaneously without complications [4,7]. Identification and removal of potential triggering factors, along with high-dose proton pump inhibitor therapy, are considered key components of management [4,7,13,14]. In patients with suspected autoimmune involvement or an inadequate response to acid suppression therapy, a personalized therapeutic approach incorporating systemic corticosteroids or other immunosuppressive agents may be considered [8,14].

In the present case, the severe mucosal involvement and histologic blistering pattern prompted empiric treatment with systemic corticosteroids. However, direct immunofluorescence and follow-up endoscopic findings were unavailable at the time of manuscript revision. Consequently, neither an autoimmune bullous etiology nor a steroid-related endoscopic response could be confirmed.

The nutritional strategy was individualized. Severe oral intolerance, recurrent vomiting, persistent dysphagia, and substantial unintentional weight loss led the nutrition support team to select temporary TPN. Although enteral nutrition is generally preferred whenever feasible, poor tolerance and severe mucosal injury limited its continued use. This approach should not be interpreted as a general recommendation for EDS.

This case has important limitations. First, direct immunofluorescence was unavailable, preventing definitive confirmation of autoimmune bullous disease. Second, follow-up endoscopic findings were not available at the time of manuscript revision; therefore, treatment efficacy, mucosal healing, and steroid response could not be confirmed. Third, detailed nutritional parameters, including body mass index and complete nutritional assessment values, were not available in the current records, limiting objective quantification of nutritional status beyond the documented 13 kg weight loss and clinical oral intolerance.

The principal contribution of this case lies in its diagnostic features as follows: an atypical presentation in a young patient, an apparent double-lumen configuration initially interpreted as a structural abnormality, and histopathologic findings that raised but did not confirm the possibility of autoimmune bullous involvement. These findings reinforce the need for multidisciplinary interpretation integrating endoscopy, pathology, imaging, and clinical evolution.

## Conclusions

Esophagitis dissecans superficialis is a rare and underrecognized esophageal disorder that may present with striking endoscopic findings and a broad clinical spectrum. This case highlights an atypical and severe presentation in a young patient without classic risk factors, in whom EDS mimicked a structural esophageal abnormality and led to diagnostic uncertainty. Accurate diagnosis requires careful correlation between clinical presentation, imaging findings, endoscopic features, and histopathologic evaluation, together with consideration of relevant differential diagnoses, including inflammatory, infectious, structural, neoplastic, and autoimmune bullous disorders.

Early recognition of the characteristic endoscopic features of EDS is essential to avoid misdiagnosis and unnecessary invasive procedures. In severe presentations, management should be individualized through multidisciplinary assessment. Because follow-up endoscopic evidence was unavailable in this case, no conclusions regarding the efficacy of corticosteroids, temporary esophageal rest, or parenteral nutrition can be drawn. Further studies are needed to better define diagnostic criteria and management strategies for this uncommon condition.
